# Supplementary material for: Genome-wide analyses of 200,453 individuals yield new insights into the causes and consequences of clonal hematopoiesis
Source: Nat Genet. 2022 Jul 14;54(8):1155–66. doi: 10.1038/s41588-022-01121-z (PMC9355874; doi:10.1038/s41588-022-01121-z)
Supplement: Supplementary file 1 — Reporting Summary [file 41588_2022_1121_MOESM1_ESM.pdf]

nature portfolio

Corresponding author(s): George S. Vassiliou, Siddhartha P. Kar, Pedro M. Quiros

Last updated by author(s): May 11, 2022

Reporting Summary

Nature Portfolio wishes to improve the reproducibility of the work that we publish. This form provides structure for consistency and transparency in reporting. For further information on Nature Portfolio policies, see our Editorial Policies and the Editorial Policy Checklist.

Statistics

For all statistical analyses, confirm that the following items are present in the figure legend, table legend, main text, or Methods section.

n/a	Confirmed	
☐	☒	The exact sample size (*n*) for each experimental group/condition, given as a discrete number and unit of measurement
☐	☒	A statement on whether measurements were taken from distinct samples or whether the same sample was measured repeatedly
☐	☒	The statistical test(s) used AND whether they are one- or two-sided<br>*Only common tests should be described solely by name; describe more complex techniques in the Methods section.*
☐	☒	A description of all covariates tested
☐	☒	A description of any assumptions or corrections, such as tests of normality and adjustment for multiple comparisons
☐	☒	A full description of the statistical parameters including central tendency (e.g. means) or other basic estimates (e.g. regression coefficient) AND variation (e.g. standard deviation) or associated estimates of uncertainty (e.g. confidence intervals)
☐	☒	For null hypothesis testing, the test statistic (e.g. *F*, *t*, *r*) with confidence intervals, effect sizes, degrees of freedom and *P* value noted<br>*Give P values as exact values whenever suitable.*
☒	☐	For Bayesian analysis, information on the choice of priors and Markov chain Monte Carlo settings
☒	☐	For hierarchical and complex designs, identification of the appropriate level for tests and full reporting of outcomes
☐	☒	Estimates of effect sizes (e.g. Cohen's *d*, Pearson's *r*), indicating how they were calculated

Our web collection on statistics for biologists contains articles on many of the points above.

Software and code

Policy information about availability of computer code

Data collection	Data were collected by the UK Biobank resource. We downloaded the data from the UK Biobank repository using helper programs (ukb_conv, ukbfetch, ukbgene, ukb_md5, and ukb_unpack) for the whole exome sequencing and phenotype data.

Data analysis

We used publicly available software and web-based tools for analyses: somatic variant calling was performed using Mutect2 GATK v4.1.8.1 for the discovery whole exome sequences and using Mutect2 GATK v4.2.2.0 for the replication whole exome sequences (https://github.com/broadinstitute/gatk/releases). Genome-wide association analyses were conducted using BOLT-LMM v2.3.6 (https://alkesgroup.broadinstitute.org/BOLT-LMM/BOLT-LMM_manual.html). LDSC v1.0.1 (https://github.com/bulik/ldsc) was used for heritability estimation and cell type-specific enrichment analyses. Genetic correlations were computed using HDL v1.4.0 (https://github.com/zhenin/HDL). LD-clumping was undertaken using FUMA v1.3.6b (https://fuma.ctglab.nl). Conditional analysis was carried out using the GCTA-COJO tool (https://yanglab.westlake.edu.cn/software/gcta/#COJO) in GCTA v1.93 (https://yanglab.westlake.edu.cn/software/gcta/#Download). Gene-level association analyses were performed using the MAGMA v1.08 tool (https://ctg.cncr.nl/software/magma) in FUMA v1.3.6b (https://fuma.ctglab.nl). Transcriptome-wide association analyses were conducted using SMR v1.03 (https://yanglab.westlake.edu.cn/software/smr/#Download). Protein-protein interaction network and pathway analyses were performed using NetworkAnalyst v3.0 (https://www.networkanalyst.ca). Fine-mapping was conducted using PICS2 v2.1.1 (https://pics2.ucsf.edu). Fine-mapped variant-gene body overlap and fine-mapped variant ATAC-seq peak overlap and ATAC-RNA count correlations were performed using the scripts at https://github.com/sankaranlab/mpn-gwas. SIFT and PolyPhen scores were obtained via the SNPnexus v4 (https://snp-nexus.org). Lead variants were searched on the Open Targets Genetics (https://genetics.opentargets.org) and PhenoScanner V2 (http://www.phenoscanner.medschl.cam.ac.uk) platforms. Druggability of prioritized functional target gene products was evaluated using the Open Targets Platform (https://platform.opentargets.org) and canSAR v1.5.0 (https://cansarblack.icr.ac.uk). For the observational analyses, we used the glm and p.adjust (in the R stats package v4.0.2), coxph (in the R survival package v3.2-11) and crr (in the R cmprsk package v2.2-10) functions, all implemented in R v4.0.2. For the Mendelian randomization analyses, we used the TwoSampleMR v0.5.6 R package implemented in R v4.0.5. Custom code was written for variant calling (and made available as a Nextflow v20.07.1 pipeline) and Mendelian randomization and these scripts are available at https://doi.org/10.5281/zenodo.6419042 (https://github.com/siddhartha-kar/clonal-hematopoiesis).

For manuscripts utilizing custom algorithms or software that are central to the research but not yet described in published literature, software must be made available to editors and reviewers. We strongly encourage code deposition in a community repository (e.g. GitHub). See the Nature Portfolio guidelines for submitting code & software for further information.

Data

Policy information about availability of data

All manuscripts must include a data availability statement. This statement should provide the following information, where applicable:

- Accession codes, unique identifiers, or web links for publicly available datasets
- A description of any restrictions on data availability
- For clinical datasets or third party data, please ensure that the statement adheres to our policy

Summary statistics for the overall and subtype-specific CH genome-wide association analyses reported here have been made publicly available at https://doi.org/10.5281/zenodo.5893861. They can also be downloaded from the GWAS Catalog (https://ftp.ebi.ac.uk/pub/databases/gwas/summary_statistics/GCST90102001-GCST90103000/) using the study accession numbers GCST90102618 (overall CH), GCST90102619 (DNMT3A-CH), GCST90102620 (TET2-CH), GCST90102621 (small clone CH), and GCST90102622 (large clone CH). Individual-level UK Biobank data can be requested via application to the UK Biobank (https://www.ukbiobank.ac.uk). The CH call set has been returned to the UK Biobank to enable individual-level data linkage for approved UK Biobank applications. Pre-computed 1000 Genomes phase 3 European ancestry reference panel and cell-type group LD scores used for heritability estimation and cell-type group partitioned heritability analysis, respectively, can be downloaded from https://alkesgroup.broadinstitute.org/LDSCORE. The STRING protein-protein interaction and Reactome pathway databases are available from, and were accessed via, the NetworkAnalyst 3.0 platform (https://www.networkanalyst.ca) and are also downloadable separately from http://version10.string-db.org/cgi/download.pl for STRING v10 and https://reactome.org/download-data for Reactome. Cis-expression quantitative trait locus data from the eQTLGen consortium used for the SMR analyses can be downloaded from https://www.eqtlgen.org/cis-eqtls.html. All summary genetic association statistics data sets used in the Mendelian randomization (MR) and MR-phenome-wide association analyses are publicly available at the Integrative Epidemiology Unit (IEU) OpenGWAS project portal (https://gwas.mrcieu.ac.uk/datasets) and can be accessed by entering the identifiers provided in the “id/link” column in Supplementary Table 35 and the “id.outcome” column in Supplementary Tables 41 and 42 in the “GWAS ID” field at https://gwas.mrcieu.ac.uk/datasets.

Field-specific reporting

Please select the one below that is the best fit for your research. If you are not sure, read the appropriate sections before making your selection.

☒ Life sciences ☐ Behavioural & social sciences ☐ Ecological, evolutionary & environmental sciences

For a reference copy of the document with all sections, see nature.com/documents/nr-reporting-summary-flat.pdf

Life sciences study design

All studies must disclose on these points even when the disclosure is negative.

Sample size

We used all participants (n = 200,453; age range: 38-72, 55% females) in the UK Biobank for whom whole exome sequencing data have been released in December 2020 for the primary analyses. Replication genetic association analyses were based on an additional 221,285 European-ancestry individuals (age range: 39-73, 53% females) in the UK Biobank for whom whole exome sequencing was performed after our UK Biobank discovery set. No statistical method was used to predetermine sample size.

Data exclusions

For the discovery genome-wide association analyses, we excluded participants with genetic sex mismatches, participants having non-European ancestries (by self-report or inferred by genetics) or excess heterozygosity (>3 standard deviations from the mean), and included only one of each set of related participants (third-degree relatives or closer). We also performed a targeted association analysis focused on the seven genome-wide significant ($P<5e-8$) lead variants associated with CH in European-ancestry individuals, evaluating their associations in 505 individuals with CH and 11,893 controls without CH who had diverse (non-European) ancestry and were excluded from the initial discovery genome-wide association analyses. Details are provided in the Methods section of the manuscript. The exclusion criteria were not pre-specified.

Replication	Replication was undertaken using independent somatic mutation calling and germline association analysis pipelines on data from 221,285 European-ancestry individuals in the UK Biobank, for whom whole exome sequencing was performed after our UK Biobank discovery set. We focused on DNMT3A and/or TET2 mutation carriers (n=9,386) in the replication sample, stratified by these two genes and clone size, and evaluated the 20 unique lead variants that we identified in the discovery GWAS (representing 26 distinct lead variants for overall/subtype-specific CH associations). Eighteen of 20 variants were replicated at P<0.05, with 16 of the 20 replicating at the Bonferroni threshold of P<0.05/20 and 19 of 20 showing directionally consistent effect size point estimates (Supplementary Table 22). The overall CH variant rs13130545 (4q35.1-ENPP6) and the small clone-specific variant rs72755524 (5p13.3-LINC02064) did not show an association at P<0.05 in the replication analysis. Notably, we confirmed our observation from the discovery GWAS that lead variant alleles at the TCL1A and CD164 loci also had opposite effects on DNMT3A- and TET2-CH in the replication set, and we also replicated the CHEK2 rare variant association.<br><br>Further, the following results also attest to the quality of our data and the validity of our genetic discoveries: replication at P<5e-8 of all previously reported CH risk loci identified in populations of European ancestry at genome-wide significance (P<5e-8), consistency of genome-wide SNP heritability estimates (3.6%) with previously reported estimates of narrow-sense (additive) heritability for CH, demonstration of enrichment of the genome-wide SNP association signal from our analysis in epigenetic marks specific to the hematopietic system, correlation between genome-wide association data for susceptibility to CH and mosaic chromosomal alterations (mCAs; a trait related to CH), including the fact that 13 out of the 19 CH lead variants (P<5e-8) identified by us were associated at P<1e-4 with risk of mCAs in a previously reported GWAS of mCAs (all details, including references, are in the Results section of the manuscript).
Randomization	The experiments were not randomized. Age at baseline, sex, whole-exome sequencing batch, and first 10 genetic principal components were adjusted for as covariates in BOLT-LMM models for genome-wide association analyses. For observational epidemiological analyses, regression and survival models were adjusted for age, sex, smoking status, whole-exome sequencing batch, and the first ten genetic principal components. In addition, models involving blood cell counts and traits were also adjusted for assessment center while those involving cholesterol and cholesterol species were also adjusted for cholesterol-lowering medication use.
Blinding	The experiments were not randomized and investigators were not blinded during the experiments and outcome assessment. The analyses presented in this manuscript do not include any experimental intervention or clinical trial. The investigators involved were not blind to clonal hematopoiesis "case" and control status since genetic association analyses require knowledge of this status.

Reporting for specific materials, systems and methods

We require information from authors about some types of materials, experimental systems and methods used in many studies. Here, indicate whether each material, system or method listed is relevant to your study. If you are not sure if a list item applies to your research, read the appropriate section before selecting a response.

Materials & experimental systems

n/a	Involved in the study	
☒	☐	Antibodies
☒	☐	Eukaryotic cell lines
☒	☐	Palaeontology and archaeology
☒	☐	Animals and other organisms
☐	☒	Human research participants
☒	☐	Clinical data
☒	☐	Dual use research of concern

Methods

n/a	Involved in the study	
☒	☐	ChIP-seq
☒	☐	Flow cytometry
☒	☐	MRI-based neuroimaging

Human research participants

Policy information about studies involving human research participants

Population characteristics	The UK Biobank is a prospective longitudinal study containing in-depth genetic and health information from half a million UK participants. The primary analyses in this study were based on all participants (n = 200,453; age range: 38-72, 55% females) in the UK Biobank for whom whole exome sequencing data have been released in December 2020. Replication genetic association analyses were based on an additional 221,285 European-ancestry individuals (age range: 39-73, 53% females) in the UK Biobank for whom whole exome sequencing was performed after our UK Biobank discovery set.
Recruitment	As stated above, the UK Biobank is a prospective longitudinal study containing in-depth genetic and health information from half a million UK participants. Details of UK Biobank participant recruitment are available at: https://www.ukbiobank.ac.uk and from Sudlow C, et al. (2015) PLoS Med 12(3): e1001779. For this study, we have selected 200,453 individuals who had whole-exome sequencing (WES) data released as of December 2020. Notably, participants were not selected in any way, however, as is the case for several such cohorts, there is some evidence of selection bias in favor of healthier, older, female, and socio-economically better off volunteers (Fry A, et al. Am. J. of Epidemiol., Vol. 186, Issue 9, 1 Nov. 2017, Pgs. 1026–1034.). Also, despite a relatively low response rate to invitations to participate, it has been shown that risk factor associations identified in the UK Biobank are generalizable (Batty GD, et al. BMJ 2020; 368:m131).
Ethics oversight	The UK Biobank resource was approved by the North West Multi-centre Research Ethics Committee under reference number 21/NW/0157 and all participants provided written informed consent to participate. Participants in the UK Biobank resource are volunteers and not compensated for participation. Data from the UK Biobank resource were accessed under approved application numbers 56844, 29202, and 26041 for this study. Further details can be found at: https://www.ukbiobank.ac.uk/learn-more-about-uk-biobank/about-us/ethics.

March 2021

Note that full information on the approval of the study protocol must also be provided in the manuscript.
